# Special issue on protein acetylation: from molecular modification to human disease

**DOI:** 10.1038/s12276-018-0103-4

**Published:** 2018-07-27

**Authors:** Eun ji Lee, Ji Hae Seo, Kyu-Won Kim

**Affiliations:** 1 College of Pharmacy and Research Institute of Pharmaceutical Sciences, Seoul National University, Seoul, 08826 Korea; 2 Department of Biochemistry, Keimyung University School of Medicine, Daegu, 42601 Korea; 3 Crop Biotechnology Institute, GreenBio Science and Technology, Seoul National University, Pyeongchang, 25354 Korea

Protein acetylation is one of the major post-translational modifications (PTMs) that regulates various biological processes in eukaryotic cells. By transferring the acetyl functional group (CH_3_CO–) from acetyl coenzyme A (Ac-CoA) to a specific site of a polypeptide chain, eukaryotes modulate cellular proteins to perform essential regulatory roles in diverse cellular pathways. Two distinct forms of protein acetylation are normally observed: N-terminal (Nt) modification and lysine (K) modification. Nt-acetylation was initially thought to be co-translational since the Nα-termini of 80–90% of human nascent polypeptides were reported to be modified in this manner. However, recent studies have identified a post-translational form of this modification, revealing the complex system of Nt-acetylation. Both co-translational and post-translational reactions are catalyzed by the highly conserved Nt-acetyltransferases (NATs) and are considered irreversible, since no corresponding Nt-deacetylases have been identified. This modification is especially important for deciding the fate of proteins, such as protein lifespan, folding, and subcellular localization, and the misregulation of these processes results in various pathological consequences, including neurodegenerative diseases, tumor development, and other human disorders.

On the other hand, K acetylation is a reversible modification mediated by K acetyltransferases (KATs) at the ε-amino group of K. This reaction was first observed in the nucleus, where counteractive interplay between histone acetyltransferases (HATs) and histone deacetylases (HDACs) resulted in dynamic transitions in chromatin condensation, resulting in transcriptional regulation. Later studies reported the broad subcellular distribution of various acetylated proteins, suggesting that the K acetylation is a global PTM, regulating not only transcription but also other important cellular processes. Today, HATs are recognized as KATs, and their corresponding HDACs are recognized as K deacetylases (KDACs); the tight regulation of K acetylation carried out by these enzymes plays fundamental regulatory roles in development, cancer progression, and other diverse human disorders.

As recent findings improve our knowledge of acetyltransferases and the molecular consequences of their action, the need for an integrated understanding of protein acetylation is emerging. In this special issue, we offer a series of representative perspectives on protein acetylation in different molecular and pathological fields that are provided by leaders on each topic. Authors mainly focus on N-alpha-acetyltransferase 10 (Naa10), also known as arrest-defective 1 protein (ARD1), as it is the only enzyme that is reported to exhibit both Nt and K acetyltransferase activities. A comparison of its dual mode-of-action will provide a deeper understanding of protein acetylation to readers.

To first discuss the Nt-acetylation activity of Naa10/ARD1, Arnesen and his colleagues provide an overview of eukaryotic Nt-acetylation by NATs and its impacts. This paper highlights how this modification affects protein fate, including stability, folding, protein–protein interactions, and subcellular targeting, in eukaryotic cells. Along with the nature of the enzymatic machinery of NAT complexes and their cellular roles, they also outline techniques to study Nt-acetylation. In addition, Hwang and his team specifically focus on the control of protein degradation by the N-end rule pathway, which is triggered by Nt-acetylation. Their paper reviews how Nt-acetylation regulates the in vivo half-life of proteins by associating with various components of the protein degradation machinery, providing a novel understanding of intracellular proteolysis control by this modification.

For K acetylation activity, Seo and her colleagues discuss its molecular mechanisms and biological implications. After summarizing the major families of KATs and KDACs, this paper proposes regulatory mechanisms of ARD1 that alter its K acetyltransferase activity. Moreover, it demonstrates various molecular consequences of ARD1-misregulation and their cellular and pathological importance.

Moving on to the pathological perspective, Naa10/ARD1 is related to various diseases, including developmental disorders and cancer progression. Lyon and his colleague review a broad range of “NAA10-related disorders”, which cover an intellectual disability named Ogden syndrome, developmental delay, and cardiac dysmorphisms found in humans. They present the steps for establishing a diagnosis, provide a clinical description, and discuss the related molecular mechanisms of these disorders. In detail, Oh’s group specifically focuses on the role of Naa10 in the development of various multicellular organisms, including humans, mice, and zebrafish. They summarize the findings on the expression and functions of Naa10 during embryonic development and emphasize the importance of future studies on cell-specific activities and distinct pathways of Naa10.

Overexpression or mutation of ARD1 is known to promote cancer progression and lead to a poor prognosis in various cancer types, including breast, lung, intestinal cancer, and stomach carcinomas. Chung and his colleagues suggest a correlation between ARD1 and hepatocellular carcinoma (HCC), a representative of malignancies with a poor outcome. They provide molecular mechanisms of ARD1-mediated tumorigenesis and clinical implications in HCC, guiding the development of specific biomarkers for the early detection and prognosis prediction of this cancer. On the other hand, Liu’s group focuses on prostate cancer. Androgen receptor (AR) plays critical roles in prostate cancer (PCa) development, and acetylation of this protein by ARD1 leads to its activation, resulting in PCa progression. This paper provides a comprehensive understanding of the ARD1-AR acetylation axis and its importance as a potential anti-cancer target in PCa.

Since its first discovery approximately 50 years ago, protein acetylation has emerged as an important PTM that is essential for numerous cellular metabolic pathways in eukaryotes. Despite its long history, most of the remarkable observations about this modification were made in the past 20 years, leaving much more to be explored. These 7 papers introduce the world of acetylation, from its basic concepts to its biological impact on humans. We hope that this special issue will be a milestone in the exciting journey of protein acetylation as well as a good resource for its overall understanding.

